# Antifungal Activity of Homoaconitate and Homoisocitrate Analogs

**DOI:** 10.3390/molecules171214022

**Published:** 2012-11-27

**Authors:** Maria J. Milewska, Marta Prokop, Iwona Gabriel, Marek Wojciechowski, Sławomir Milewski

**Affiliations:** 1 Department of Organic Chemistry, Gdańsk University of Technology, 11/12 Narutowicza Str., 80-233 Gdańsk, Poland; 2 Department of Pharmaceutical Technology and Biochemistry, Gdańsk University of Technology, 11/12 Narutowicza Str., 80-233 Gdańsk, Poland

**Keywords:** homoisocitrate, homoaconitate, synthesis, inhibitors, molecular modeling, antifungal activity

## Abstract

Thirteen structural analogs of two initial intermediates of the l-α-aminoadipate pathway of l-lysine biosynthesis in fungi have been designed and synthesized, including fluoro- and epoxy-derivatives of homoaconitate and homoisocitrate. Some of the obtained compounds exhibited at milimolar range moderate enzyme inhibitory properties against homoaconitase and/or homoisocitrate dehydrogenase of *Candida albicans*. The structural basis for homoisocitrate dehydrogenase inhibition was revealed by molecular modeling of the enzyme-inhibitor complex. On the other hand, the trimethyl ester forms of some of the novel compounds exhibited antifungal effects. The highest antifungal activity was found for trimethyl *trans*-homoaconitate, which inhibited growth of some human pathogenic yeasts with minimal inhibitory concentration (MIC) values of 16–32 μg/mL.

## 1. Introduction

Microbial resistance to antibiotics and synthetic drugs is an emerging challenge for clinicians and the pharmaceutical industry. The multidrug-resistant fungi are the major cause of failure in chemotherapy of disseminated mycoses [1,2]. Thus, the need for novel antifungals is especially urgent. Among different strategies of searching for new potential drugs, identification of novel, unexploited molecular targets and their inhibitors seems one of the most promising approaches. These days many researchers are concentrated on fungal enzymes that catalyze essential biochemical reactions, specific to pathogen cells, and absent from the host cells. Bioactive compounds that selectively block metabolic pathways in human pathogenic fungi could be potential antifungals with low mammalian toxicity.

l-Lysine is an essential amino acid for humans, while bacteria, plants and fungi have developed pathways of lysine biosynthesis. There are two versions of these anabolic routes: a diaminopimelic acid pathway, which is characteristic for bacteria, plants and lower fungi and an α-aminoadipate pathway (AAP) for higher fungi, *Ascomycetes* and *Basidomycetes*, including most of the human pathogenic yeasts and filamentous fungi. Enzymes that catalyze the reactions of the above mentioned first pathway are considered promising molecular targets for antibacterial chemotherapy [3], while the second pathway could be a source of new targets for antifungal chemotherapy [4]. Diminished virulence in some systems of fungal cells lacking genes encoding enzymes of AAP was demonstrated [5,6], and the antifungal activity of compounds targeting one of these enzymes was shown too [7].

In this paper we present results of our studies on the design, synthesis and evaluation of biological properties of some novel structural analogs of homoaconitate and homoisocitrate, intermediates of the AAP pathway.

## 2. Results and Discussion

**Scheme 1:**
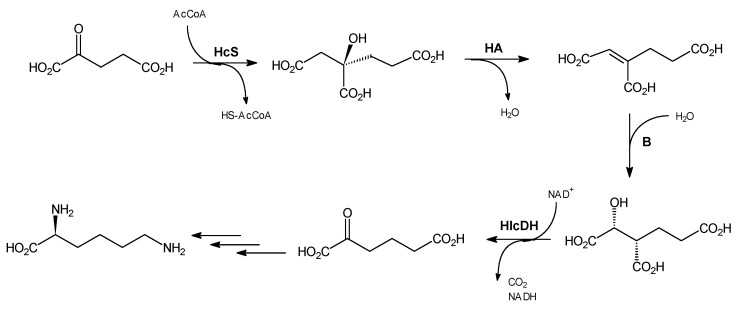
Details of the three initial reactions of AAP. HcS—homocitrate synthase; HA—homoaconitase; HIcDH—homoisocitrate dehydrogenase.

Three initial reactions of the AAP (Scheme 1) are catalyzed by the enzymes that are unique to fungi, so that their selective inhibitors may have antifungal potential. We have designed and synthesized several compounds that are structural analogs of substrates/products of two of the enzymes, homoaconitase (HA) and homoisocitrate dehydrogenase (HIcDH), *i.e.*, *cis*-homoaconitate and homoisocitrate. Whenever possible, the compounds that are di- or tricarboxylic acids were obtained in two forms, potassium/sodium salts or methyl esters. It was expected that the ester forms of potential enzyme inhibitors should better penetrate the fungal cell membrane than their salt counterparts and after internalization could be cleaved by intracellular esterases.

The following compounds were synthesized: *cis*-homoaconitate analogs: *trans*-homoaconitates **1a** and **2**, methyl ester and potassium salts of *trans*-1,2-epoxybutane-1,2,4-tricarboxylate (compounds **3** and **4**); homoisocitrate analogs: (2*S*,3*R*)-2-hydroxy-3-propylsuccinate (**5a**); (2*S*,3*R*)-2-hydroxy-3-butylsuccinate (**5b**); (2*S*,3*R*)-2-hydroxy-3-allylsuccinate (**5c**); (2*R*,3*S*)-2-fluoro-3-propylsuccinate (**6a**); (2*R*,3*S*)-2-fluoro-3-butylsuccinate (**6b**); (2*R,*3*S*)-2-fluoro-3-allylsuccinates **6c** and **7c**; (2*R*,3*S*)-2-fluoro-2-deoxyhomoisocitrates **8**–**10**.

### 2.1. Synthesis of Homoaconitate and Homoisocitrate Analogs

**Scheme 2:**
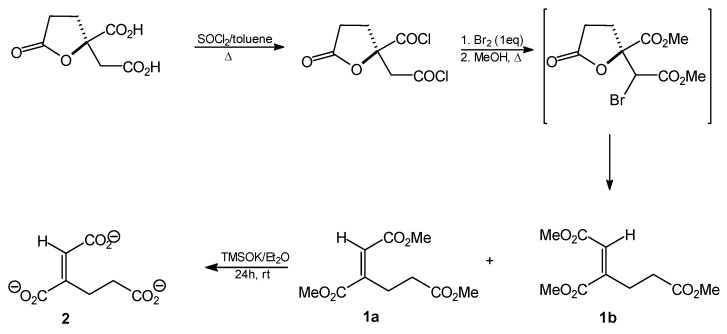
Synthetic route to compounds **1** and **2**.

Two stereoisomers of homoaconitate were synthesized from (*R*)-homocitric lactone following the strategy shown in Scheme 2. The lactone was first converted into the respective dichloride. This compound was then brominated at the α-methylene position. Heating in methanol promoted the conversion of the acid chlorides into methyl esters, ring opening and HBr elimination. In this way, a mixture of *cis* and *trans* isomers of homoaconitate (molar ratio 3:1) was obtained with the overall yield of 37%. The stereoisomers could be effectively separated by flash chromatography. Both were easily, almost quantitatively converted into respective tripotassium salts upon treatment with potassium trimethylsilanolate.

**Scheme 3:**
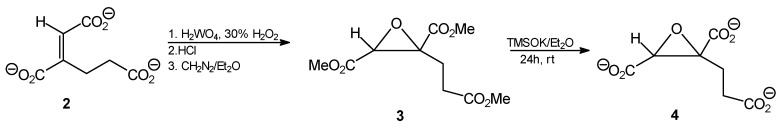
Synthesis of epoxy derivatives **3** and **4**.

Treatment of *trans*-homoaconitate with H_2_WO_4_/H_2_O_2_, as shown in Scheme 3, afforded corresponding *trans*-epoxide in high yield. 

Rather unexpectedly, the same methodology applied to tripotassium *cis*-homoaconitate as a substrate, resulted in formation of *trans*-epoxide as the only product in 33% yield. The stereochemistry of this compound was unequivocally confirmed by comparison of its ^1^H- and ^13^C-NMR spectra to those of reference samples of *cis*- and *trans*-1,2-epoxy-propane-1,2,3-carboxylate.

**Scheme 4:**
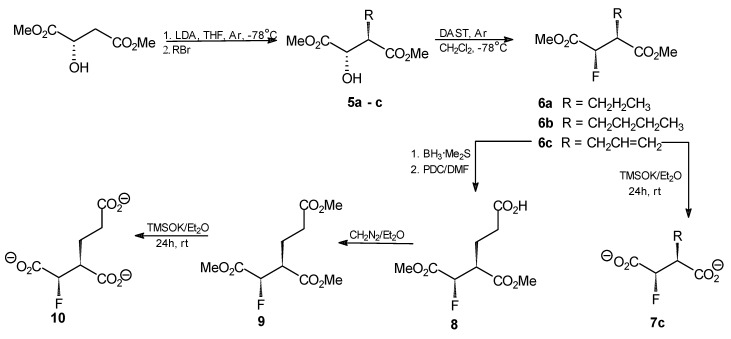
Strategy of synthesis of deoxyfluoro homoisocitrate analogs **5**–**10**.

Exchange of the hydroxyl substituent for fluorine for the purpose of preparation of deoxyfluoro analogs of homoisocitrate was performed by use of *N*,*N*-(diethylamino)sulfur trifluoride (DAST) as fluorinating agent. Surprisingly, the reaction in which homoisocitrate was a substrate, afforded a mixture of homoaconitate stereoisomers as the only products. Therefore, an alternative multistep strategy shown in Scheme 4 was applied. 

Dimethyl (*S*)-malate was first alkylated with an appropriate alkyl/allyl bromide. This reaction was highly stereoselective, as expected for a Seebach-type alkylation [8] and resulted in formation of single diastereoisomers of compounds **5a**–**c**. The stereochemistry of these compounds was assigned as (2*S*,3*R*), based on the known stereospecificity of the Seebach alkylation of (*S*)-malate [8,9]. With dimethyl (*R*)-malate as a substrate, (2*R*,3*S*) stereoisomers were formed as the only products (data not shown). Treatment of compounds **5a**–**c** with DAST afforded single diastereoisomers of the succinate derivatives **6a**–**c**. This reaction, as a nucleophilic substitution of the S*_N_*2 type [10], lead to the inversion of configuration at the hydroxyl-substituted C2 atom. Obviously, there was no inversion of the actual absolute configuration at C3 in this reaction but its stereochemical notation changed, due to the introduction of fluorine at C2. Thus the (2*R*,3*S*) configuration could be assigned to compounds **6a**–**c**. Compound **6c** was converted into dimethyl (2*R*,3*S*)-2-fluoro-2-deoxyhomoisocitrate in a two-step reaction. The trimethyl ester **9** was obtained upon treatment of dimethyl (2*R*,3*S*)-2-fluoro-2-deoxyhomoisocitrate **8** with an ethereal solution of diazomethane and could be converted into the respective tripotassium salt **10**. All the compounds **1**–**10** were fully characterized using their spectroscopic data.

### 2.2. Biological Activity of Novel Compounds

Compounds **1**–**10** were tested for their *in vitro* antifungal activity against six yeast strains. MICs of the studied compounds determined by the microplate serial dilution method are shown in Table 1. None of the salts (compounds **2**, **4**, **7c**, **10**) showed any antifungal activity. The highest activity was found for trimethyl *trans*-homoaconitate (**1a**) that inhibited the growth of all tested yeast strains, with MIC values in the 16–32 μg/mL range. The corresponding epoxide **3** inhibited growth of four out of the six yeast strains, but its MIC values were much higher (128–512 μg/mL). Among the deoxyfluoro analogs of homoisocitrate, some antifungal activity against selected strains was noted for dimethyl (2*R*,3*S*)-2-fluoro-3-allylsuccinate (**6c**) and trimethyl (2*R*,3*S*)-2-fluoro-2-deoxyhomoisocitrate (**9**) (MICs 64-512 μg/mL). In all cases the MIC values were higher than those determined for the known antifungal fluconazole, however *trans*-homoaconitate **1a** was only slightly less effective.

It was expected that the novel compounds in the free acid or salt form could inhibit activity of homoaconitase or homoisocitrate dehydrogenase. To check this possibility, the effect of compounds **1**–**10** on activity of recombinant homoaconitase and homoisocitrate dehydrogenase from *Candida albicans* was quantified. According to the expectations, none of the ester derivatives: **1a**, **3**, **5a**–**c**, **6a**–**c** and **9** had any effect on enzyme activity at concentrations up to 10 mM. Effects noted for salts **2**, **4**, **7c** and **10** are summarized in Table 2.

*Trans*-homoaconitate **2** and the epoxy derivative **4** inhibited homoaconitase, while the 2-fluoro-2-deoxy analogs of homoisocitrate, *i.e.*, compounds **7c** and **10**, affected the activity of homoisocitrate dehydrogenase. The IC_50_ values were in the milimolar range and as such were much higher than those of the most active inhibitors of homoisocitrate dehydrogenase known so far that inhibited the *S. cerevisiae* enzyme at micromolar concentrations [9,10]. Nevertheless, one can clearly see a correlation between the enzyme inhibitory properties of compounds **2**, **4**, **7c** and **10** and the antifungal activity of their respective ester derivatives **1a**, **3**, **6** and **9**. It is very likely therefore that the ester compounds penetrate the fungal cell membrane and are metabolized intracellularly to release the active enzyme inhibitors. Such a correlation has been demonstrated for the first time for inhibitors of the AAP. The previous reports concerned either enzymatic inhibitors without any data on their antifungal properties [11,12], or described antifungal action of methyl esters of carboxyalkyl- and carboxyaryl-substituted D-malic acid derivatives without identification of any enzymatic target [7].

### 2.3. Molecular Modeling of Enzyme: Inhibitor Interactions

**Figure 1:**
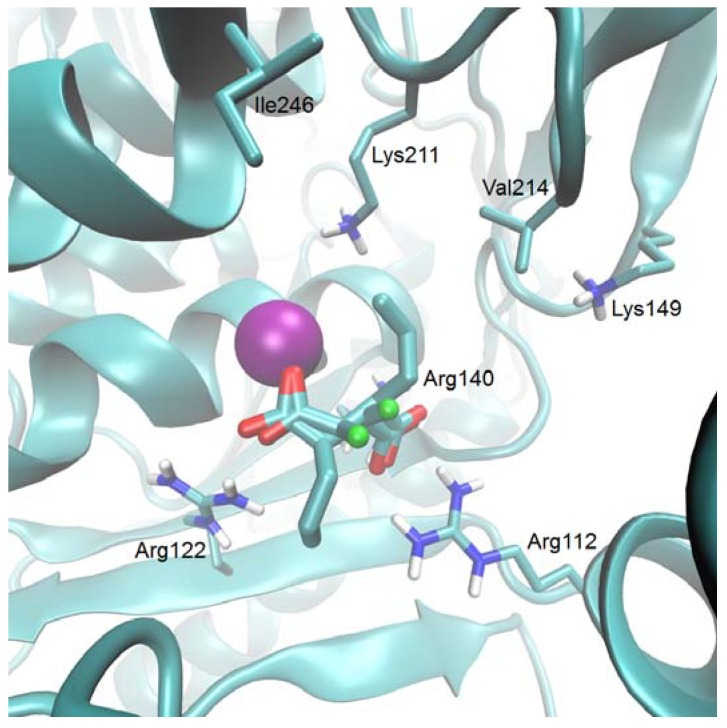
Compound **7c** docked at the active site of modeled CaHIcDH. The most probable two orientations of the ligand are shown. Mg(II) ion of the active site is displayed as a violet sphere and a fluorine atom of **7c** is presented as a green ball.

Compounds **7c** and **10** are structural analogs of homoisocitrate. Despite the fact that the real 3D arrangement of respective substituents of the C3 atom of these compounds is opposite to that in the HIcDH dehydrogenase physiological substrate, *i.e.*, in (2*R*,3*S*)-homoisocitrate (despite the identity of their formal stereochemical notations), they both inhibit enzyme activity. In order to elucidate a molecular basis for this inhibition, we have studied a pattern of interactions of **7c** docked into the modeled *C. albicans* HIcDH active site. This active centre is in fact the only site for inhibitor binding, since in HIcDH there are no other specific ligand binding sites, including those for possible allosteric inhibitors [13]. Results of these studies are shown in Figure 1. 

The C1 carboxylate of the inhibitor is bound to the active site magnesium ion, while the C7 carboxylate is bound by the receptor’s Arg112 and Arg140 guanidine moieties. The divalent cations Arg112 and Arg140 were previously identified as those involved in binding of the respective carboxylate functions of the homoisocitrate substrate [13,14]. On the other hand, in the most probable orientation, the allyl substituent at C3 of **7c** points toward the small, partially hydrophobic pocket formed by the Val214, Ile246 residues and the nonpolar part of the Lys211 side chain. This pocket is supposed to be the binding site of the NADH substrate [13]. The hydrophobic character of the niche accommodating the C3 substituent explains the slightly higher inhibitory potential of **7c** (the allyl derivative) in comparison to **10**, bearing the carboxylate function. It should be noted therefore, that the third carboxylate function of (2*R*,3*S*)-homoisocitrate docked at the active site of *C. albicans* HIcDH is bound by the ε-amino group of Lys149 [14], so that one may assume that binding of **7c** should be weaker than that of the substrate, due to the substitution of relatively strong electrostatic interactions between carboxylate of the substrate and Lys149 by the much weaker hydrophobic effect. Nevertheless, binding of **7c** and **10** at the active site at the orientation partially overlapping that of the homoisocitrate substrate, seems the most plausible reason of its weak CaHIcDH inhibitory properties.

## 3. Experimental

### 3.1. Materials and Reagents

Solvents and reagents were purchased from Sigma-Aldrich, St. Louis, MO. USA. Melting points are uncorrected. NMR spectra were recorded on the Varian Gemini 200 and 500 MHz spectrometers, using TMS as an internal standard in ^1^H and ^13^C experiments and CFCl_3_ in ^19^F experiments. Thin layer chromatography (TLC) was performed on silica gel plates (Merck, Darmstadt, Germany) using the following solvent systems as eluents: (A) hexane/ethyl acetate (9:5); (B) benzene/ethyl acetate/ethanol (85:20:5). Elementary analysis (EA) was run on a Carlo Erba, type EA 1108 analyzer. Optical rotations were measured on a Rudolph Autpol II polarimeter at 589 nm.

### 3.2. Chemical Synthesis

Trisodium (*R*)-homocitrate, dimethyl (S)-methylenehomocitrate and (*R*)-homocitric lactone were synthesized as described previously [15]. (2*R*,3*S*)-Homoisocitric acid was synthesized according to the procedure of Ma and Palmer [9].

*Trimethyl homoaconitate* (**1a** and **1b**). Thionyl chloride (9.6 mL; 132 mmol) was added dropwise to a solution of (*R*)-homocitric lactone (13.5 g; 66 mmol) in toluene (400 mL) and the resulting mixture was heated under reflux for 48 h. The solvent was then removed by evaporation under reduced pressure to give the corresponding dichloride as an oily residue (13 g; 54 mmol; 82%). Anhydrous bromine (3 mL; 54 mmol) was then added dropwise and the mixture was heated under reflux for 1 h. An additional portion of bromine (1 mL) was then added and heating was continued overnight. Anhydrous methanol (500 mL) was added to the cooled reaction mixture and the solution was heated under reflux for about 4 h. The solvent was then distilled off, the residue was dissolved in ethyl acetate and sequentially washed with aqueous 2% Na_2_CO_3_ solution, water, 3% Na_2_CO_3_ solution and water. The organic layer was then dried over anhydrous MgSO_4_ and the solvent was subsequently removed by evaporation *in vacuo*. The crude oily product (5.27 g, 37%, mixture of *cis* and *trans* isomers) was resolved by silica gel (70–230 mesh) column chromatography. The column was developed with hexane/AcOEt (5:1). The following oily products were isolated: trimethyl *trans*-homoaconitate (**1a**, 1.12 g, 8%) and trimethyl *cis*-homoaconitate (**1b**, 3.54 g, 24.5%).

*Trimethyl trans-homoaconitate* [*trimethyl (1E)-but-1-ene-1,2,4-tricarboxylate*, **1a**]. R_f_ = 0.54 (solvent system A). ^1^H-NMR (CDCl_3_) δ [ppm]: 2.54 (*t*, *J* = 7.9 Hz, 2H, CH_2_CH_2_CO); 3.09 (*t*, *J* = 7.7 Hz, 2H, CH=CCH_2_); 3.67 (*s*, 3H, OCH_3_); 3.78 (*s*, 3H, OCH_3_); 3.82 (*s*, 3H, OCH_3_); 6.82 (*s*, 1H, =CH-); ^13^C-NMR (CDCl_3_) δ[ppm]: 23.58; 33.28; 51.92; 52.14; 52.91; 127.94; 146.12; 166.01; 167.10; 173.08. Elemental anal. (%), calcd. for C_10_H_14_O_6_: C, 52.17; H, 6.09; found: C, 52.14; H, 5.93.

*Trimethyl cis-homoaconitate* [*trimethyl (1Z)-but-1-ene-1,2,4-tricarboxylate* **1b**]. R_f_ = 0.47 (solvent system A). ^1^H-NMR (CDCl_3_) δ [ppm]: 2.55 (*t*, *J* = 7.2 Hz, 2H, CH_2_CH_2_CO); 2.69 (*t*, *J* = 7.5 Hz, 2H, CH=CCH_2_); 3.69 (s, 3H, OCH_3_); 3.73 (s, 3H, OCH_3_); 3.83 (s, 3H, OCH_3_); 5.90 (*s*, 1H, =CH-); ^13^C-NMR (CDCl_3_) δ [ppm]: 29.16; 31.49; 51.86; 51.91; 52.44; 120.86; 147.67; 165.21; 168.51; 172.08. Elemental anal. (%), calcd. for C_10_H_14_O_6_: C, 52.17; H, 6.09; found: C, 52.07; H, 5.97.

*Tripotassium trans-homoaconitate* [*tripotassium (1E)-but-1-ene-1,2,4-tricarboxylate* **2**]. Trimethyl *trans*-homoaconitate (**1a**, 276 mg, 1.2 mmol) was dissolved in anhydrous diethyl ether (20 mL). Potassium trimethylsilanolate (510 mg, 4 mmol) was added to the solution and the mixture was stirred for 24 h. The solvent was removed by evaporation under reduced pressure, the residue was washed with anhydrous diethyl ether and finally dried under reduced pressure. The product was obtained as a slightly brownish powder (322 mg, 89%). ^1^H-NMR (D_2_O) δ [ppm]: 2.54 (*t*, *J* = 7.9 Hz, 2H, CH_2_,); 2.74 (*t*, *J* = 8.1 Hz, 2H, CH_2_,); 6.51 (s, CH=C, 1H). ^13^C-NMR (D_2_O) δ [ppm]: 23.54; 33.32; 51.82; 52.18; 52.99; 127.90; 146.02. Elemental anal. (%), calcd. for C_7_H_5_O_6_K_3_: C, 27.63; H, 2.30; found: C, 27.69; H, 2.27.

*Trimethyl (1E)1,2-epoxy-butane-1,2,4-tricarboxylate* (**3**). Tripotassium *trans*-homoaconitate (**2**, 302 mg; 1 mmol) was dissolved in water (5 mL) and tungstic acid (35 mg; 0.14 mmol) and 30% H_2_O_2_ (300 μL) were added. The mixture was stirred for 2 h at 85 °C. Then 10 M HCl (0.35 mL) was added and the reaction mixture was subjected to continuous extraction with diethyl ether for several hours. The organic solvent was removed from the extract by evaporation, to give 172 mg of a slightly yellowish oil. The ethereal solution of diazomethane was then added dropwise at 0 °C until pH of the solution reached 7.0, then the mixture was left overnight at room temperature. After solvent evaporation, 216 mg of the crude oily product was obtained, that was further purified by column silica gel chromatography eluting with hexane/AcOEt (5:1), followed by AcOEt. Finally, after solvent evaporation, a slightly yellow oily product was obtained (202 mg; 82%); R_f_ = 0.57 (solvent system B). ^1^H-NMR (CDCl_3_) δ [ppm]: 2.39–2.33 (m, 1H, CH_2_CH_2_CO); 2.63–2.53 (m, 2H, CH_2_CH_2_CO); 2.75–2.67 (m, 1H, CH_2_CH_2_CO); 3.67 (s, 3H, OCH_3_); 3.69 (s, 3H, OCH_3_); 3.70 (s, 3H, OCH_3_); 3.74 (s, 1H, CH); ^13^C-NMR (CDCl_3_) δ [ppm]: 31.57; 45.64; 52.19; 52.92; 52.97; 53.21; 60.05; 168.16; 170.82; 173.23. Elemental anal. (%), calcd. for C_10_H_14_O_7_: C, 48.78; H, 5.69; found: C, 48.67; H, 5.63.

*Tripotassium (1E)1,2-epoxy-butane-1,2,4-tricarboxylate* (**4**). Trimethyl (1*E*)1,2-epoxybutane-1,2,4-tricarboxylate (**3**, 148 mg, 0.6 mmol) was dissolved in anhydrous diethyl ether (10 mL). Potassium trimethylsilanolate (255 mg, 2 mmol) was added to the solution and the mixture was stirred for 24 h. The solvent was removed by evaporation under reduced pressure, the residue was washed with anhydrous diethyl ether and finally dried under reduced pressure. The product was obtained as a yellow amorphous powder (156 mg, 82%). ^1^H-NMR (D_2_O) δ [ppm]: 2.29–2.35 (m, 1H, CH_2_CH_2_CO); 2.57–262 (m, 2H, CH_2_CH_2_CO); 2.66–2.71 (m, 1H, CH_2_CH_2_CO); 3.78 (s, 1H, CH); ^13^C-NMR (CDCl_3_) δ [ppm]: 31.32; 45.68; 51.88; 53.15; 53.24; 53.48; 61.60. Elemental anal. (%), calcd. for C_7_H_5_O_7_K_3_: C, 27.27; H, 1.62; found: C, 27.27; H, 1.65.

*General procedure for the synthesis of compounds* **5a**–**c***. n*-BuLi (20.8 mL, 52.2 mmol, 2.5 M in hexane) was added dropwise to a stirred solution of diisopropylamine (62.6 mmol) in THF (38 mL) at −78 °C under argon. After 1 h, a solution of dimethyl (*S*)-malate (3.38 g, 20.9 mmol) in THF (6 mL) was added, while the temp. was kept at −78 °C. The mixture was then warmed to −25 °C and stirred for 1 h. After that time, the mixture was again chilled to −78 °C and propyl, butyl or allyl bromide (53 mmol) was added dropwise. The resulting solution was stirred for 2 h at −25 °C and then left overnight at 4 °C. The mixture was chilled to −78 °C, acetic acid (6.4 mL in 10 mL of Et_2_O) was added and the resulting mixture was combined with Et_2_O (250 mL) and water (30 mL). The organic layer was washed with the saturated solution of sodium carbonate and with saline. Aqueous layers were combined and extracted three times with Et_2_O. Combined ethereal layers were dried over anhydrous MgSO_4_ and the solvent was evaporated. The crude products were purified by flash silica-gel chromatography with hexane/AcOEt (5:1) as an eluent to afford the final products.

*Dimethyl (2S,3R)-2-hydroxy-3-propylsuccinate* (**5a**). Oil, final yield 58%. [α${]}_{D}^{20}$ +19.7 (c1.33; CHCl_3_). ^1^H-NMR (CDCl_3_) δ [ppm]: 0.95 (*t*, 3H, CH_2_CH_2_CH_3_), 1.41 (*m*, 2H, CH_2_CH_2_CH_3_), 1.66 (*m*, 1H, CH_2_CH_2_CH_3_), 1.83 (*m*, 1H, CH_2_CH_2_CH_3_), 3.69 (*s*, 3H, OCH_3_); 3.80 (s, 3H, OCH_3_); 4.29 (*d*, *J* = 3.9 Hz, 1H, CHOH); ^13^C-NMR (CDCl_3_) δ [ppm]: 14.08; 20.79; 30.36; 48.54; 52.18; 52.93; 71.22; 173.74; 174.18. Elemental anal. (%), calcd. for C_9_H_16_O_5_: C, 52.94; H, 7.84; found: C, 52.87; H, 7.90.

*Dimethyl (2S,3R)-2-hydroxy-3-butylsuccinate* (**5b**). Oil, final yield 65%. [α${]}_{D}^{20}$ +12.8 (c1.15; CHCl_3_). ^1^H-NMR (CDCl_3_) δ [ppm]: 0.94 (*t*, 3H, CH_2_CH_2_CH_2_CH_3_), 1.39 (*m*, 2H, CH_2_CH_2_CH_2_CH_3_), 1.68 (*m*, 2H, CH_2_CH_2_CH_2_CH_3_), 1.89 (*m*, 1H, CH_2_CH_2_CH_2_CH_3_), 3.70 (*s*, 3H, OCH_3_); 3.77 (s, 3H, OCH_3_); 4.29 (*d*, *J* = 4.2 Hz, 1H, CHOH); ^13^C-NMR (CDCl_3_) δ [ppm]: 12.01; 18.45; 21.01; 30.63; 48.91; 51.65; 52.98; 70.88; 173.11; 174.55. Elemental anal. (%), calcd. for C_10_H_18_O_5_: C, 55.05; H, 8.26; found: C, 54.98; H, 8.30.

*Dimethyl (2S,3R)-2-hydroxy-3-allylsuccinate* (**5c**). Oil, final yield 62%. [α${]}_{D}^{20}$ +16.1 (c1.24; CHCl_3_). ^1^H-NMR (CDCl_3_) δ [ppm]: 2.45 (*m*, 1H, CH_2_CH=CH_2_); 2.63 (*m*, 1H, CH_2_CH=CH_2_); 2.99 (*m*, 1H, CH(OH)CHCO_2_CH_3_); 3.69 (*s*, 3H, OCH_3_); 3.81 (*s*, 3H, OCH_3_); 4.31 (*d*, *J* = 3 Hz, 1H, CH(OH)CHCO_2_CH_3_); 5.19–5.11 (*dd*, *J* = 17 Hz and 10 Hz, 2H, CH_2_CH=CH_2_); 5.82 (*m*, 1H, CH_2_CH=CH_2_); ^13^C-NMR (CDCl_3_) δ [ppm]: 32.36; 48.38; 52.27; 52.99; 70.36; 118.30; 134.91; 172.78; 174.21. Elemental anal. (%), calcd. for C_9_H_14_O_5_: C, 53.47; H, 6.93; found: C, 53.37; H, 7.01.

*General procedure for the synthesis of compounds* **6a**–**c***.* DAST (0.76 mL, 5.9 mmol) was dissolved in anhydrous methylene chloride (30 mL) and the resulting solution was chilled to −78 °C under argon. An analogously chilled solution of one of the compounds **5a**–**c** (4.4 mmol) in methylene chloride (10 mL) was added dropwise using a cannulated syringe and the resulting mixture was stirred for 1 h at −78°C and for 2 h at room temperature. Chloroform (40 mL) was added and the resulting solution was washed with 5% NaHCO_3_, water and saline. The solvent was evaporated and the crude products were purified by flash silica-gel chromatography with hexane/AcOEt (9:1) to afford the final products.

*Dimethyl (2R,3S)-2-fluoro-3-propylsuccinate* (**6a**). Oil, final yield 92%. [α${]}_{D}^{20}$ +6.2 (c1.5; CHCl_3_). ^1^H-NMR (CDCl_3_) δ [ppm]: 0.93 (*t*, 3H, CH_2_CH_2_CH_3_), 1.32 (*m*, 1H, CH_2_CH_2_CH_3_), 1.43 (*m*, 1H, CH_2_CH_2_CH_3_), 1.62 (*m*, 1H, CH_2_CH_2_CH_3_), 1.82 (*m*, 1H, CH_2_CH_2_CH_3_), 2.98 (*m*, 1H, FCHCHCO_2_CH_3_), 3.74 (*s*, 3H, OCH_3_); 3.82 (*s*, 3H, OCH_3_); 5.16 (*dd*, *^2^J*_HF_ = 47.4 Hz, *^3^J*_HH_ = 5.9 Hz, 1H, FCH); ^13^C-NMR (CDCl_3_) δ [ppm]: 14.07; 20.67; 29.30; 47.91 (*^2^J*_FC_ = 23.8 Hz); 52.43; 52.85; 88.92 (*J*_FC_ = 197.3 Hz); 169.04; 172.21. ^19^F-NMR (CDCl_3_) δ[ppm]: −197.51 (*dd*, ^3^*J*_HF_ = 23 Hz, ^2^*J*_HF_ = 47 Hz). Elemental anal. (%), calcd. for C_9_H_15_O_4_F: C, 52.43; H, 7.28; found: C, 52.34; H, 7.35.

*Dimethyl (2R,3S)-2-fluoro-3-butylsuccinate* (**6b**). Oil, final yield 88%. [α${]}_{D}^{20}$ +4.9 (c1.3; CHCl_3_). ^1^H-NMR (CDCl_3_) δ [ppm]: 0.93 (*t*, 3H, CH_2_CH_2_CH_2_CH_3_), 1.24 (*m*, 1H, CH_2_CH_2_CH_2_CH_3_), 1.32 (*m*, 1H, CH_2_CH_2_CH_2_CH_3_), 1.41 (*m*, 1H, CH_2_CH_2_CH_2_CH_3_), 1.53 (*m*, 1H, CH_2_CH_2_CH_2_CH_3_), 1.62 (*m*, 1H, CH_2_CH_2_CH_2_CH_3_); 1.82 (*m*, 1H, CH_2_CH_2_CH_2_CH_3_); 2.99 (*m*, 1H, FCHCHCO_2_CH_3_), 3.74 (*s*, 3H, OCH_3_); 3.82 (*s*, 3H, OCH_3_); 5.17 (*dd*, 1H, ^2^*J*_HF_ = 42 Hz, ^3^*J*_HH_ = 5.9 Hz, FCH); ^13^C-NMR (CDCl_3_) δ [ppm]: 12.01; 18.45; 21.01; 30.63; 48.91; 51.65; 52.98; 70.88; 173.11; 174.5514.07; 20.67; 29.30; 47.91 (*^2^J*_FC_ = 23.8 Hz); 52.43; 52.85; 88.92 (*J*_FC_ = 197.3 Hz); 169.04; 172.21. ^19^F-NMR (CDCl_3_) δ [ppm]: −197.46 (*dd*, ^3^*J*_HF_ = 19.2 Hz and ^2^*J*_HF_ = 46.8 Hz). Elemental anal. (%), calcd. for C_10_H_17_O_4_F: C, 54.55; H, 7.73; found: C, 54.48; H, 7.73.

*Dimethyl (2R,3S)-2-fluoro-3-allylsuccinate* (**6c**). Oil, final yield 89%. [α${]}_{D}^{20}$ +2.5 (c1.7; CHCl_3_). ^1^H-NMR (CDCl_3_) δ [ppm]: 2.46 (*m*, 1H, CH_2_CH=CH_2_); 2.60 (*m*, 1H, CH_2_CH=CH_2_); 3.10 (*m*, 1H, CH(OH)CHCO_2_CH_3_), 3.73 (*s*, 3H, OCH_3_); 3.83 (*s*, 3H, OCH_3_); 5.11 (*m*, 1H, FCHCHCO_2_CH_3_); 5.18 (*m*, 1H, CH_2_CH=CH_2_); 5.24 (*m*, 1H, CH_2_CH=CH_2_); 5.76 (m, 1H, CH_2_CH=CH_2_); ^13^C-NMR (CDCl_3_) δ [ppm]: 38.21; 47.74 (^2^*J*_FC_ = 21.5 Hz); 52.13; 52.62; 88.20 (*J*_FC_ = 190.2 Hz); 119.57; 133.08; 165.68; 169.06; ^19^F NMR (CDCl_3_) δ[ppm]: -198.16 (*dd*, ^3^*J*_HF_ = 21 Hz; ^2^*J*_HF_ = 47 Hz). Elemental anal. (%), calcd. for C_9_H_13_O_4_F: C, 52.94; H, 6.37; found: C, 53.02; H, 6.40.

*Dipotassium (2R,3S)-2-fluoro-3-allylsuccinate* (**7c**). Dimethyl (2*R,*3*S*)-2-fluoro-3-allylsuccinate (**6c**, 41 mg; 0.2 mmol) was dissolved in anhydrous diethyl ether (20 mL). Potassium trimethylsilanolate (56 mg, 0.44 mmol) was added to the solution and the mixture was stirred for 24 h. The solvent was removed by evaporation under reduced pressure, the residue was washed with anhydrous diethyl ether and finally dried under reduced pressure. The product was obtained as a slightly yellow amorphous powder (35 mg; 70%). ^1^H-NMR (CDCl_3_) δ [ppm]: 2.44 (*m*, 1H, CH_2_CH=CH_2_); 2.59 (*m*, 1H, CH_2_CH=CH_2_); 3.14 (*m*, 1H, CH(OH)CHCO_2_CH_3_), 5.11 (*m*, 1H, FCHCHCO_2_CH_3_); 5.20 (*m*, 1H, CH_2_CH=CH_2_); 5.25 (*m*, 1H, CH_2_CH=CH_2_); 5.75 (m, 1H, CH_2_CH=CH_2_); ^13^C-NMR (CDCl_3_) δ[ppm]: 38.44; 47.07 (^2^*J*_FC_ = 23 Hz); 52.35; 53.62; 88.20 (*J*_FC_ = 192 Hz); 118.22; 135.69; ^19^F-NMR (CDCl_3_) δ [ppm]: −197.55 (*dd*, ^3^*J*_HF_ = 18 Hz; ^2^*J*_HF_ = 44 Hz). Elemental anal. (%), calcd. for C_7_H_7_O_4_FK_2_: C, 33.33; H, 2.78; found: C, 33.39; H, 2.79.

*Dimethyl (2R,3S)-2-fluoro-2-deoxyhomoisocitrate* (8). BH_3_ × SMe_2_ (0.51 mL, 5.05 mmol) was added dropwise to a solution of dimethyl (2*R*,3*S*)-2-fluoro-3-allyl succinate (6c, 1.03 g, 5.05 mmol) in methylene dichloride (8 mL) at 0 °C. The reaction mixture was left at room temperature with stirring for 2 h. Then pyridinium dichromate (PDC, 14.6 g, 38 mmol) in anhydrous DMF (30 mL) was slowly added and stirring was continued for 48 h. After that time, the mixture was transferred to the flask containing water (300 mL) and the whole solution was stirred for another 16 h. The resulting mixture was extracted with diethyl ether (3 × 250 mL) and the aqueous layer was further extracted with ethyl acetate (2 × 170 mL). The organic layers were combined and dried over anhydrous MgSO_4_. The drying agent was removed and the organic solvents were evaporated under reduced pressure to give 1.2 g of the crude product that was further purified by flash chromatography on silica gel with stepwise elution with hexane/AcOEt (5:1), AcOEt and MeOH to give 0.87 g (73%) of the purified product. ^1^H-NMR (CDCl_3_) δ [ppm]: 2.02 (*m*, 1H, CH_2_CH_2_COOH); 2.21 (*m*, 1H, CH_2_CH_2_COOH); 2.48 (*m*, 2H, CH_2_CH_2_COOH ); 3.03 (*m*, 1H, FCHCOOMe); 3.76 (*s*, 3H, OCH_3_); 3.83 (*s*, 3H, OCH_3_); 5.32–5.22 (*dd*, *^3^J*_HH_ = 4.4 Hz, *^2^J*_HF_ = 47.4 Hz, CHF); ^13^C-NMR (CDCl_3_) δ [ppm]: 23.59; 31.80; 47.77; 51.98; 52.34; 53.09; 172.66; 173.52. ^19^F-NMR (CDCl_3_) δ [ppm]: −196.23 (*dd*, *^3^J*_HF_ = 21 Hz; *^2^J*_HF_ = 47 Hz). Elemental anal. (%), calcd. for C_9_H_13_O_6_F: C, 45.76; H, 5.50; found: C, 45.71; H, 5.47.

*Trimethyl (2R,3S)-2-fluoro-2-deoxyhomoisocitrate* [*trimethyl (1R,2S)-1-fluorobutane-1,2,4-tricarboxylate*, **9**]. Dimethyl (2*R*,3*S*)-2-fluoro-2-deoxyhomoisocitrate (**8**, 203 mg, 0.9 mmol) was treated with diazomethane solution in diethyl ether under argon. The mixture was left overnight at room temperature. The solvent was then removed by evaporation under reduced pressure. The residue was washed twice with diethyl ether. The crude oily product was purified by flash chromatography on silica gel with hexane/AcOEt (2:1) as an eluent. The final product was obtained as a colorless oil (228 mg, 95%). [α${]}_{D}^{20}$ +12.5 (c2.0; CHCl_3_). ^1^H-NMR (CDCl_3_) δ [ppm]: 2.04 (*m*, 1H, CH_2_CH_2_COOMe); 2.21 (*m*, 1H, CH_2_CH_2_COOMe); 2.48 (*m*, 2H, CH_2_CH_2_COOMe); 3.03 (*m*, 1H, FCHCOOMe); 3.71 (*s*, 3H, OCH_3_); 3.72 (*s*, 3H, OCH_3_); 3.83 (s, 3H, OCH_3_); 4.63 (*d*, *J* = 3.4 Hz, 1H, FCH); ^13^C-NMR (CDCl_3_) δ [ppm]: 23.59; 31.80; 47.77; 51.98; 52.34; 53.09; 71.34; 172.66; 173.52; 173.84. ^19^F-NMR (CDCl_3_) δ [ppm]: −193.44 (*dd*, *^3^J*_HF_ = 22 Hz; *^2^J*_HF_ = 49 Hz). Elemental anal. (%), calcd. for C_10_H_15_O_6_F: C, 50.00; H, 6.25; found: C, 50.07; H, 6.22.

*Tripotassium (2R,3S)-2-fluoro-2-deoxyhomoisocitrate* [*tripotassium (1R,2S)-1-fluorobutane-1,2,4-tricarboxylate*, **10**]. Conversion of the trimethyl ester **9** into the respective tripotassium salt was achieved upon treatment with potassium trimethylsilanolate under condition described above for preparation of compound **4**. The product was obtained as a yellowish amorphous powder (309 mg, 93%). ^1^H-NMR (D_2_O) δ [ppm]: 2.02 (*m*, 1H, CH_2_CH_2_COOK); 2.25 (*m*, 1H, CH_2_CH_2_COOK); 2.52 (*m*, 2H, CH_2_CH_2_COOK); 3.01 (*m*, 1H, FCHCOOK); 4.68 (*d*, *J* = 3.4 Hz, 1H, FCH); ^13^C-NMR (CDCl_3_) δ [ppm]: 23.48; 31.85; 47.72; 51.95; 52.38; 53.00; 71.31. ^19^F-NMR (CDCl_3_) δ[ppm]: −192.58 (*dd*, *^3^J*_HF_ = 20 Hz; *^2^J*_HF_ = 51 Hz). Elemental anal. (%), calcd. for C_7_H_6_O_6_FK_3_: C, 48.58; H, 4.86; found: C, 48.51; H, 4.92.

### 3.3. Determination of Antifungal Activity

Antifungal *in vitro* activity was determined by the modified M27-A3 method specified by the CLSI [16] in RPMI-1640 medium buffered to pH 7.0. The test micro-organisms were: *Candida albicans* ATCC 10231, *Candida glabrata* DSM 11226, *Candida tropicalis* KKP 334 *Candida krusei* DSM 6128, *Candida lusitaniae* DSM 70102 and *Saccharomyces cerevisiae* ATCC 9763. Wells containing serially diluted examined compounds and compound-free controls were inoculated with 12 h cultures of tested strains to the final concentration of 10^4^ colony-forming units (cfu)/mL. Plates were incubated for 24 h at 37 °C and growth was then quantified by measuring an optical density at 531 nm, using a microplate reader (Victor^3^V; Perkin Elmer, Centre of Excellence ChemBioFarm). The MIC was defined as the lowest drug concentration at which at least 80% decrease in turbidity, in comparison to the drug-free control, was observed.

### 3.4. Determination of Enzyme Inhibition by Novel Compounds

Recombinant *C. albicans* homoaconitase (HA) and homoisocitrate dehydrogenase (HIcDH) containing oligoHis *N*-terminal tags were constructed and purified to near homogeneity as described earlier [14,17,18]. Both recombinant proteins exhibited enzymatic activities identical to their wild type counterparts. Recombinant HA demonstrated K_M_ = 1.5 mM for (*R*)-homocitrate and recombinant HIcDH showed K_M_ = 74 μM for (2*R*, 3*S*)-homoisocitrate and K_M_ = 1.1 mM for NAD^+^.

Reaction catalyzed by homoisocitrate dehydrogenase (HIcDH) was monitored by measuring the NADH absorption at 340 nm. The standard assay mixture contained 10 mM KCl, 5.0 mM MgCl_2_, 5 mM NAD^+^ and 0.1 mM (2*R*,3*S*)-homoisocitrate in 50 mM Hepes-NaOH (pH 7.8) buffer. The reaction mixture with all required components except for the enzyme was preincubated for 3 min and the reaction was started by an addition of the recombinant His_6_-HIcDH (50 μg). The formation of NADH was measured for 30 s at 20 °C to determine initial reaction velocity. The initial rates were determined using a linear portion of the reaction progress curve.

Activity of homoaconitase (HA) was measured by a coupled reaction with HIcDH by monitoring the reduction of NAD^+^ to NADH at 340 nm [19]. Reaction mixtures containing 50 mM Tris-HCl (pH 8.5), 50 mM KCl, 5 mM MgCl_2_, 20 mM dithiothreitol, 1 mM NAD^+^ and 30 μg/mL of His_6_-HIcDH in a total volume of 0.9 mL were degassed under argon for 10 min, after which 0.05 mL of His_6_-HAC solution was added to a final concentration of 10 μg/mL. The reaction was initiated with addition of 0.05 mL of 50 mM (*R*)-homocitrate. The formation of NADH was measured for 30 s at 20 °C to determine initial reaction velocity. The initial rates were determined using a linear portion of the reaction progress curve. 

The effects of novel compounds on the HA and HIcDH activity was determined by measuring the enzyme activity in standard assay mixtures containing various concentration of an inhibitor (0–10 mM). Inhibitory potential was expressed as IC_50_ values, *i.e*., concentrations causing 50% inhibition of enzyme activity in relation to control. All determinations were performed in triplicate. 

### 3.5. Molecular Modeling and Docking Calculations

Model of the receptor (CaHIcDH) was built using the template of *Schisosaccharomyces pombe* HIcDH [13], as described elsewhere [12]. Docking calculations of all ligands were performed with the AutoDock software version 4.2 [20]. Initial geometries of the ligands were built by means of the Accelrys Discovery Studio. Final AutoDock topologies of ligands and receptors in the pdbqt format, including the appropriate for the program atom types and partial charges, were calculated and assigned with AutoDock accompanied scripts. Docking grid was centered in the middle of the binding site at the Arg122 guanidine moiety of HIcDH. Semi flexible docking methodology was used (with fixed receptor and flexible ligands) and Lamarckian genetic algorithm (LGA) was chosen as the search engine. The initial size of the population was set to 150 random solutions and the number of generations was limited to 27,000. To ensure the convergence of the search procedure number of objective function evaluations was raised to 25,000,000. Docking procedure was repeated 50 times and the resulting 50 ligand poses were clustered with the tolerance of 1.8 Å. The most abundant and the lowest energy clusters were selected for analysis.

## 4. Conclusions

Two analogs of *cis*-homoaconitate, namely the tripotassium salts of *trans*-homoaconitate and (1*E*)1,2-epoxybutane-1,2,4-tricarboxylate were found to inhibit activity of *C. albicans* HA, while tripotassium *(*2*R,*3*S)-*2-fluoro-3-allylsuccinate and tripotassium (2*R*,3*S*)-2-fluoro-2-deoxyhomo-isocitrate inhibited HIcDH from the same source. The trimethyl ester derivatives of these compounds inhibited growth of some human pathogenic yeast, including *C. albicans*. We have been thus able to demonstrate for the first time that inhibitors of particular enzymes catalyzing initial steps of fungi-specific AAP pathway may exhibit antifungal activity after conversion into more lipophilic, membrane-permeable forms. The compounds described in this work may serve as leads for further studies on the design of compounds targeting AAP as potential antifungals. In light of our results, it seems very likely that the ester forms of strong inhibitors of the enzymes of AAP may exhibit good antifungal potential, so that a further search for such compounds as potential antifungal pro-drugs is undoubtedly worth pursuing.

## Conflict of Interests

The authors declare no conflict of interests.

## Figures and Tables

**Table 1:** Antifungal *in vitro* activity of compounds **1**–**10**.

Comp.	MIC (μg/mL)					
	*S. cerevisiae*	*C. albicans*	*C. glabrata*	*C. tropicalis*	*C. lusitaniae*	*C. krusei*
**1a**	32	16	16	32	16	32
**2**	>512	>512	>512	>512	>512	>512
**3**	>512	256	128	128	512	>512
**4**	>512	>512	>512	>512	>512	>512
**5a**	>512	>512	>512	>512	>512	>512
**5b**	>512	>512	>512	>512	>512	>512
**5c**	>512	>512	>512	>512	>512	>512
**6a**	>512	>512	>512	>512	512	>512
**6b**	>512	256	512	128	128	>512
**6c**	>512	256	256	256	>512	512
**7c**	>512	>512	>512	>512	512	>512
**9**	>512	128	256	64	>512	256
**10**	>512	>512	>512	>512	>512	>512
Flu *	4	4	8	2	4	8

* Flu = Fluconazole.

**Table 2:** Enzyme inhibitory potential of compounds **2**, **4**, **7c** and **10**. Values are the means of three independent determinations ± SD.

Compound	IC_50_ (mM)	
	HA	HIcDH
**2**	1.58 ± 0.11	> 10 mM
**4**	5.76 ± 0.34	> 10 mM
**7c**	>10 mM	6.78 ± 0.56
**10**	>10 mM	3.22 ± 0.55
